# Imaging Kv1.3 Expressing Memory T Cells as a Marker of Immunotherapy Response

**DOI:** 10.3390/cancers14051217

**Published:** 2022-02-26

**Authors:** Julian L. Goggi, Shivashankar Khanapur, Boominathan Ramasamy, Siddesh V. Hartimath, Tang Jun Rong, Peter Cheng, Yun Xuan Tan, Xin Yi Yeo, Sangyong Jung, Stephanie Shee Min Goay, Seow Theng Ong, You Yi Hwang, K. George Chandy, Edward G. Robins

**Affiliations:** 1 Institute of Bioengineering and Bioimaging (IBB), Agency for Science, Technology and Research (A*STAR), 11 Biopolis Way, #01-02 Helios, Singapore 138667, Singapore; shivashankar@ibb.a-star.edu.sg (S.K.); boominathan_ramasamy@ibb.a-star.edu.sg (B.R.); s_hartimath@ibb.a-star.edu.sg (S.V.H.); tang_jun_rong@ibb.a-star.edu.sg (T.J.R.); peter_cheng@ibb.a-star.edu.sg (P.C.); tan_yun_xuan@hq.a-star.edu.sg (Y.X.T.); edward_robins@ibb.a-star.edu.sg (E.G.R.); 2 Department of Physiology, Yong Loo Lin School of Medicine, National University of Singapore, Singapore 119077, Singapore; syjung@imcb.a-star.edu.sg; 3 Institute of Molecular and Cell Biology (IMCB), Agency for Science, Technology and Research (A*STAR), 11 Biopolis Way, #01-02 Helios, Singapore 138667, Singapore; tp-yeoxy@imcb.a-star.edu.sg; 4 LKCMedicine-ICESing Ion Channel Platform, Lee Kong Chian School of Medicine, Nanyang Technological University, 59 Nanyang Drive, Singapore 636921, Singapore; sheemin.goay@ntu.edu.sg (S.S.M.G.); st.ong@ntu.edu.sg (S.T.O.); g.chandy@ntu.edu.sg (K.G.C.); 5 Singapore Immunology Network (SIgN), Agency for Science, Technology and Research (A*STAR), 8A Biomedical Grove, Immunos, Singapore 138648, Singapore; leon_hwang@immunol.a-star.edu.sg; 6 Clinical Imaging Research Centre (CIRC), Yong Loo Lin School of Medicine, National University of Singapore, 14 Medical Drive, #B1-01, Singapore 117599, Singapore

**Keywords:** immune checkpoints, positron emission tomography (PET), potassium channels

## Abstract

**Simple Summary:**

Cancer immunotherapy has shown huge potential in the fight against cancer but few patients respond durably to treatment. Durable immunological responses are associated with the generation of memory T cells. A subset of memory T cells, effector memory T cells, have been shown to be associated with tumours responding to immune checkpoint inhibitor therapy in a range of cancer types. These effector memory T cells overexpress the potassium channel, Kv1.3, on the cell surface. In the current manuscript, we have developed a new Kv1.3 targeting peptide radiopharmaceutical, [^18^F]AlF-NOTA-KCNA3P, and characterised its in vivo biodistribution and ability to stratify response to immune checkpoint inhibitor therapy, correlating tumour uptake to changes in tumour associated immune cell populations. Overall, [^18^F]AlF-NOTA-KCNA3P successfully predicted response to immune checkpoint inhibitors in a murine model of colon cancer where tracer uptake in the tumour correlated well with changes in tumour associated memory T cell populations.

**Abstract:**

Immune checkpoint inhibitors have shown great promise, emerging as a new pillar of treatment for cancer; however, only a relatively small proportion of recipients show a durable response to treatment. Strategies that reliably differentiate durably-responding tumours from non-responsive tumours are a critical unmet need. Persistent and durable immunological responses are associated with the generation of memory T cells. Effector memory T cells associated with tumour response to immune therapies are characterized by substantial upregulation of the potassium channel Kv1.3 after repeated antigen stimulation. We have developed a new Kv1.3 targeting radiopharmaceutical, [^18^F]AlF-NOTA-KCNA3P, and evaluated whether it can reliably differentiate tumours successfully responding to immune checkpoint inhibitor (ICI) therapy targeting PD-1 alone or combined with CLTA4. In a syngeneic colon cancer model, we compared tumour retention of [^18^F]AlF-NOTA-KCNA3P with changes in the tumour immune microenvironment determined by flow cytometry. Imaging with [^18^F]AlF-NOTA-KCNA3P reliably differentiated tumours responding to ICI therapy from non-responding tumours and was associated with substantial tumour infiltration of T cells, especially Kv1.3-expressing CD8^+^ effector memory T cells.

## 1. Introduction

Immune checkpoint inhibitors (ICIs) have been rapidly adopted as the standard of care in many advanced tumours, however, durable response rates are low [1,2,3,4] and the mechanisms regulating long term success for ICIs in specific cancers remains poorly understood. The infiltration of T lymphocytes (TILs) is necessary for ICI efficacy in numerous cancer types, hence biomarkers to quantify CD8^+^ expression have been developed [5,6,7,8,9] and shown to act as a useful biomarker for response clinically [10]. However, the mere presence of CD8^+^ TILs may not be enough for accurate stratification of response to ICI therapy due to the presence of suppression and resistance mechanisms in the tumour microenvironment, which can inhibit functional activation of these T cells [11,12]. Accurate stratification requires a marker of T cell activation [13].

Transmembrane ion channels are required for T cell activation after antigen presentation. Antigen driven calcium signalling during T cell activation is counterbalanced and sustained by potassium efflux through voltage-gated Kv1.3 and calcium-activated KCa3.1 potassium channels, which are expressed by quiescent T cells [14,15]. Repeated antigenic exposure differentiates T cells into memory T cells that rapidly respond to further antigen challenges. A subset of memory T cells, effector memory T cells (T_EM_), display a substantial increase in Kv1.3 expression compared to other T cells [16]. These cells have been shown to be associated with tumours responding to ICI therapy in a range of cancer types. Indeed, a recent publication by Newton et al. shows that the Kv1.3 channel is enriched among TILs in head and neck cancer patients that respond to ICI therapy with PD1 inhibitors [17]. In vitro expansion of TILs after PD1 blockade shows a significant increase in T_EM_ cells in responsive tumours versus non-responsive tumours with minor differences in CD8^+^ cells overall [18]. As T_EM_ cells are characterised by substantial upregulation of the Kv1.3 potassium channel, compared to naïve or central memory T cells [14,15], we hypothesised that Kv1.3 expression may act as an accurate measure of tumour response to ICI therapy. 

**Figure 1:**
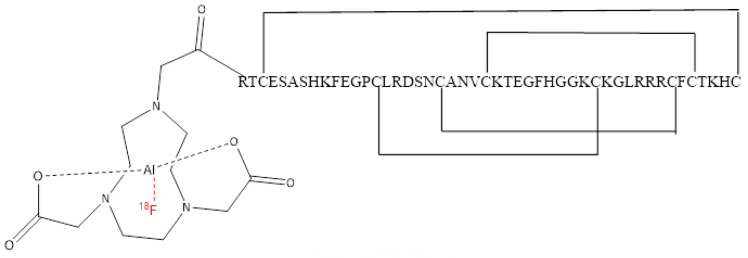
Structure of [^18^F]AlF-NOTA-KCNA3P.

We radiolabelled a previously characterised selective peptide modulator (EgK5) of Kv1.3 channels [16] to quantify Kv1.3 channel expression on tumour infiltrating T_EM_ cells and evaluated whether this novel Kv1.3-targeting peptide-radiopharmaceutical, [^18^F]AlF-NOTA-KCNA3P (Figure 1), was able to successfully stratify response to ICIs in a CT26 syngeneic colon cancer model in vivo, correlating tumour retention to tumour infiltrating immune cells using flow cytometry.

## 2. Materials and Methods

### 2.1. General Information

The precursor NOTA-KCNA3P peptide was custom synthesised by the Chinese Peptide Company (CPC) (Hangzhou, China) with >95% purity. All other materials are listed in the Supplementary Materials.

### 2.2. [^18^F]AlF-NOTA-KCNA3P Radiochemistry

[^18^F]AlF-NOTA-KCNA3P peptide was radiolabelled and purified using a modified procedure similar to other [^18^F]AlF labelling methodologies [9]. The full method is described in Supplementary Materials (Section 1.5 including Supplementary Figures S1 and S2, Tables S1 and S2). Starting from aqueous no-carrier-added [^18^F]fluoride (typically 10 GBq in 2.4 mL), [^18^F]AlF-NOTA-KCNA3P (Figure 1) was isolated as a 10% ethanol in saline formulated product in a non-decay corrected radiochemical yield of 11.9 ± 6.2, with a radiochemical purity of greater than 99% and molar activity of 75 ± 45 GBq/µmol (*n* = 8).

### 2.3. Electrophysiology Studies

The effects of the peptides NOTA-KCNA3P and EgK5 on K_V_1.2, K_V_1.3 and K_V_1.5 [19] channels were evaluated by patch-clamp as detailed in the Supplementary Materials (Supplementary Figure S3) using a QPatch HTX automated electrophysiology platform. 

### 2.4. Animal Procedures

All animal procedures strictly followed IACUC guidelines (IACUC No. 181399) and conformed to NIH guidelines and public law. Development of the animal model has been described previously [20]. Briefly, BALB/c mice aged 6–8 weeks were purchased from In Vivos (Singapore) and implanted subcutaneously into the right shoulder with the murine colon cancer cell line CT26 (ATCC, 2 × 10^5^ cells per animal 1:1 (*v*:*v*) ratio in Matrigel). Tumour volumes were repeatedly measured on days 6, 9, 12, 15, 19 and 21 post tumour implantation. The mice were randomised and dosed by intraperitoneal (i.p.) injection on days 6, 9 and 12 following tumour inoculation. Treatment groups included control (5 mg/kg, rat IgG2a isotype control, α-trinitrophenol mAb, IP), αPD1 mAb monotherapy (10 mg/kg, rat IgG2a anti-mouse PD-1, RMP1-14) and combined αPD1 and αCTLA4 mAb therapy (10 mg/kg and 5 mg/kg, respectively, mouse IgG2b anti-mouse CTLA-4, 9D9, Bio-X Cell). 

Determination of tumour response to therapy was made using tumour growth inhibition (%TGI) on day 21 as previously described [20] (Supplementary Table S5). 

### 2.5. PET-CT Imaging

Mice were imaged under isoflurane anaesthesia and injected with [^18^F]AlF-NOTA-KCNA3P (~10 MBq) via the lateral tail vein. Dynamic time–activity curves were acquired to assess tracer uptake and clearance profiles (Supplementary Figure S4) and tail vein blood samples taken to assess in vivo metabolic stability (Supplementary Figure S7). To stratify response to therapy, animals were imaged on day 12 using a Siemens Inveon PET-CT as previously described [20]. Briefly, 20 min static PET acquisitions were acquired from 60 min post-injection and analysis of the reconstructed calibrated images was performed and volumes of interest delineated by CT imaging were used to determine tissue uptake (whole body distribution shown for reference in Supplementary Figure S8). Data are expressed as % of the injected dose per gram (%ID/g) of tumour tissue in the volume of interest. 

### 2.6. Flow Cytometry

Tumours were removed after the completion of imaging and processed for flow cytometry as previously described [20]. The samples were then counted and assessed for viability with Trypan Blue (Sigma-Aldrich, St. Louis, MO, USA). Cells were stained with antibodies against Kv1.3 (polyclonal FITC; Sigma-Aldrich), CD103 (clone M290 FITC; BD Biosciences, San Jose, CA, USA), CD25 (clone PC61 BB700; BD Biosciences), CD45 (clone 30-F11 BUV395; BD Biosciences), Fixable Live/Dead Blue (Invitrogen, Waltham, MA, USA), CD62L (clone MEL-14 BUV563; BD Biosciences), CD86 (clone GL1 BUV615; BD Biosciences), F4/80 (clone T45-2342; BD Biosciences), NKp46 (clone 29A1.4 BUV737; BD Biosciences), CD3e (clone 500A2 BUV805; BD Biosciences), FoxP3 (clone 150D AlexaFluor647; Biolegend, San Diego, CA, USA), CD44 (clone IM7 APC-R700; BD Biosciences), CD11b (clone M1/70 APC-Cy7; Biolegend, San Diego, CA, USA), Granzyme B (clone QA16A02 PE; Biolegend), CCR7 (clone 4B12 PE-CF594; BD Biosciences), CD19 (clone 6D5 PE-Cy5; Biolegend), CD206 (clone C068C2 PE-Cy7; Biolegend), CD127 (clone SB/199 BV421; BD Biosciences), Ly6G (clone 1A8 BV480; BD Biosciences), CD8 (clone 53-6.7 BV510; BD Biosciences), CD11c (clone N418 BV570; Biolegend), Ly6C (clone HK1.4 BV605; Biolegend), Siglec F (clone E50-2440 BV650, BD Biosciences), CD68 (clone FA-11 BV711; Biolegend), CD4 (clone GK1.5 BV750; BD Biosciences), I-A/I-E (clone M5/114.15.2 BV785; Biolegend).

A BD FACSymphony was utilised for flow cytometry assessment of infiltrating cells and data post processed and analysed using FlowJo V10.7.1 software (FlowJo LLC, Ashland, OR, USA). 

### 2.7. Dimension Reduction Analysis

Dimension reduction analysis was performed on fcs files exported from FlowJo (time-gated, size-gated, Live, singlet, CD45 positive cells) and clustering was assessed using Rphenograph as previously described [20]. The dimension reduction was carried out using the cytofkit package in RStudio (https://github.com/JinmiaoChenLab/cytofkit, accessed on 23 February 2022). One of the inbuilt dimension reduction choices in cytofkit was t-distributed stochastic neighbour embedding (t-SNE). This statistical method gives each data point, in this case, each single cell, a location in a two-dimensional graph. The t-SNE algorithm takes the multi-dimensional flow data from each cell and constructs a probability distribution between pairs of cells such that similar objects are assigned a higher probability. This builds out the two-dimensional plot in a way that similar cells across all inputted parameters are clustered together in space. The Rphenograph is an undirected clustering method that groups similar cells into clusters based on the distance and number of nearby neighbouring cells. The default cytofkit parameters were used for the analysis on 5000 cells from each fcs file, for a total of 195,000 cells. The following markers were used for the Rphenograph clustering: Kv1.3, CD3, CD4, CD8, CD11b, CD11c, CD19, CD25, CD44, CD62L, CD206, F4/80, FoxP3, Granzyme B, I-A/I-E, Ly6C, Ly6G, Nkp46 and Siglec-F.

### 2.8. Statistical Analysis

Data were analysed using a non-parametric Kruskal–Wallis one-way ANOVA with a Dunn’s post-test to allow the comparison of multiple groups with different distributions against TNRs. All statistical assessments were performed using GraphPad Prism 8.0.0 (GraphPad Software, San Diego, CA, USA) where *p* < 0.05 was considered statistically significant. Data are expressed as the mean ± S.D. unless otherwise indicated.

## 3. Results

### 3.1. Evaluation of Treatment Efficacy Using Tumour Volumes

**Figure 2:**
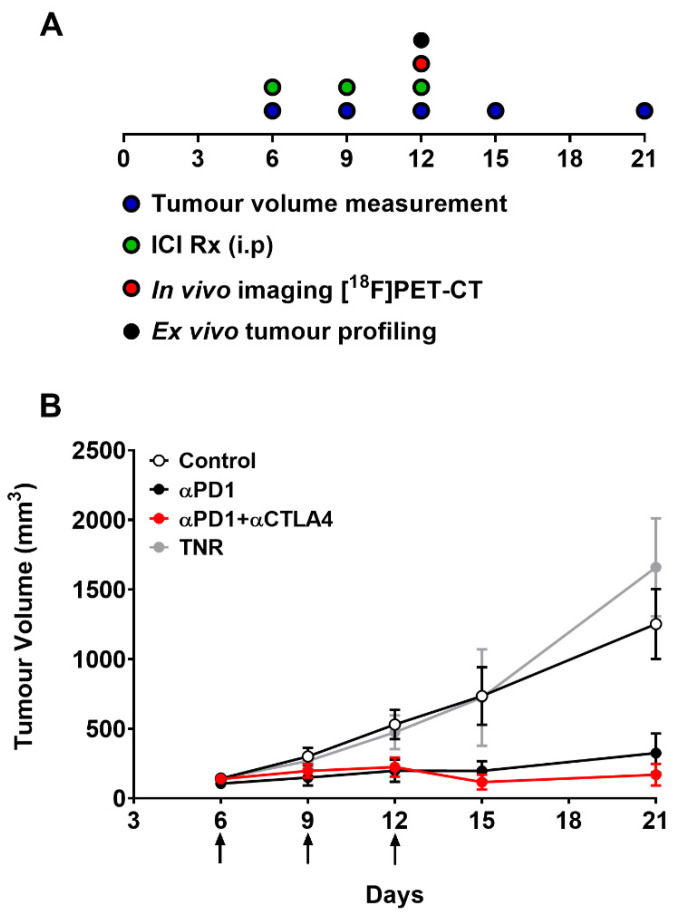
(**A**) Schematic representation of timeline showing dosing, measurement and imaging regimen. Mice (*n* = 10–15) were i.p. treated with control IgG, αPD1 monotherapy or combined αPD1 + αCTLA4 on days 6, 9, and 12 post tumour implantation. (**B**) Average tumour volume of CT26 tumour-bearing mice on days 6, 9, 12, 15, 19 and 21 post tumour implantation. Data are represented as the mean ± S.D. Data are shown post therapy response separation and represented as the mean ± S.D. (TNR, treated non-responder).

The CT26 tumour-bearing mice were subjected to the dosing and assessment regimen shown in Figure 2A. Tumour growth across the treatment arms was normally distributed (Shapiro–Wilk *p* = 0.0612) with each arm showing different treatment response rates and magnitudes depending on therapeutic intervention (Supplementary Table S4). The combined treatment arm displayed a greater response rate and magnitude than the monotherapy treatment arm. Grouped tumour volumes are shown in Figure 2B and Supplementary Table S3. Tumour growth inhibition (%TGI, Supplementary Table S5) and tumour retention of [^18^F]AlF-NOTA-KCNA3P showed good correlation before post hoc manipulation (Pearson r = 0.802, **** *p* < 0.0001, *n* = 30). The separation of treatment responsive tumours (TR, combining complete responders and partial responders) from treated non-responsive tumours (TNRs) has been described previously [20] and is based on the comparison of day 6 and day 21 tumour volumes from each individual animal. TRs are identified as those animals with final tumour volumes less than 546.4 mm^3^ (<3 SD mean volume of the control group on day 21).

### 3.2. [^19^F]AlF-Electrophysiological Characterization of NOTA-KCNA3P

EgK5, a peptide derived from a plant defensin, is a highly selective modulator of Kv1.3 [16]. EgK5 does not block Kv1.3 channels, but prolonged exposure to this peptide and its binding to the channel causes a run-down of Kv1.3 currently due to depletion of PIP_2_ and the resultant reduction in the pool of Kv1.3 channels capable of gating [16]. We used patch-clamp analysis to determine if NOTA-KCNA3P modulates Kv1.3 channels such as EgK5. We exposed L929 fibroblasts stably expressing K_V_1.3 channels overnight to NOTA-KCNA3P (0.1, 1 or 10 μM) or EgK5 (10 μM) dissolved in P6N buffer, or to media containing an equal volume of P6N buffer. NOTA-KCNA3P- and EgK5-treated cells exhibited significant run-down of Kv1.3 currents, but there was little change in current amplitude in cells exposed to media containing P6N buffer (Supplementary Figure S3A). The average current amplitude at pulses 18−20 normalized for membrane capacitance (pA/pF) showed that NOTA-KCNA3P and EgK5 reduced Kv1.3 current density compared to control cells (Supplementary Figure S3B,C). Concentration–response curves generated with either normalized current amplitude or normalized current density yielded IC_50_ (50% suppression) values of 70 nm and 50 nM, respectively, for NOTA-KCNA3P (Supplementary Figure S3D,E). To assess specificity, we examined the effect of NOTA-KCNA3P on two closely related channels, Kv1.2 and Kv1.5. No run-down of Kv1.2 and Kv1.5 currents was seen after overnight exposure to NOTA-KCNA3P (Supplementary Figure S3F,G). Taken together, our data demonstrate that NOTA-KCNAP modulates Kv1.3 with good affinity and specificity.

### 3.3. [^18^F]AlF-NOTA-KCNA3P In Vivo PET Imaging

**Figure 3:**
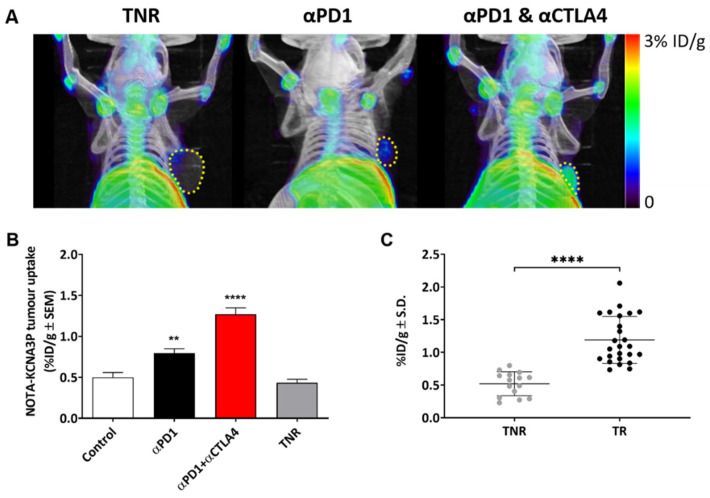
(**A**) Representative maximum intensity projection PET-CT images showing [^18^F]AlF-NOTA-KCNA3P tumour retention in αPD-1 responders, combined αPD1 + αCTLA4 responders and TNRs. Yellow dashed line indicates tumour boundary. (**B**) [^18^F]AlF-NOTA-KCNA3P tumour uptake values in each treatment arm; Control, αPD-1 responders and combined αPD1 + αCTLA4 responders compared to treated non-responsive tumours (TNR, *n* = 10–12 mice/ group; ** *p* < 0.01, **** *p* < 0.0001 comparing to TNR; data shown as the mean %ID/g ± S.E.M.). (**C**) [^18^F]AlF-NOTA-KCNA3P tumour uptake in CT26 TRs and TNRs (**** *p* < 0.0001, data shown as individual %ID/g).

Tumour uptake of [^18^F]AlF-NOTA-KCNA3P was heterogeneous depending on treatment regime and treatment efficacy (Figure 3A) with a tumour-to-blood ratio (TBR) of ~2 in tumour responders and a TBR of ~1 in tumour non-responders (TNRs). Low tumour uptake of [^18^F]AlF-NOTA-KCNA3P was measured in the TNRs and the control treatment arm. Significantly greater tumour retention was measured in the responding treatment arms (TRs); αPD1 monotherapy (* *p* < 0.05, *n* = 7) and αPD1 + αCTLA4 combination therapy (** *p* < 0.01, *n* = 10), when compared to the TNR group, *n* = 9). Tumour uptake of [^18^F]AlF-NOTA-KCNA3P was sufficient to distinguish from background and differentiated responsive tumours from treated non-responders (Table 1 and Figure 3B,C) in vivo. Ex vivo biodistribution was used to confirm the imaging results (Supplementary Figure S5). Importantly, no difference was seen between non-responders and responders in the biodistribution of [^18^F]AlF-NOTA-KCNA3P in muscle, blood, lung, skin, heart and brain, the only difference being in the tumour (Supplementary Figure S5). Furthermore, Western blot analysis of excised tumours showed that tumour response to treatment was associated with increased expression of Kv1.3 (Supplementary Figure S6).

### 3.4. Tumour Uptake of [^18^F]AlF-NOTA-KCNA3P Is Linked to Infiltration of Kv1.3 Expressing T_EM_ Cells

**Figure 4:**
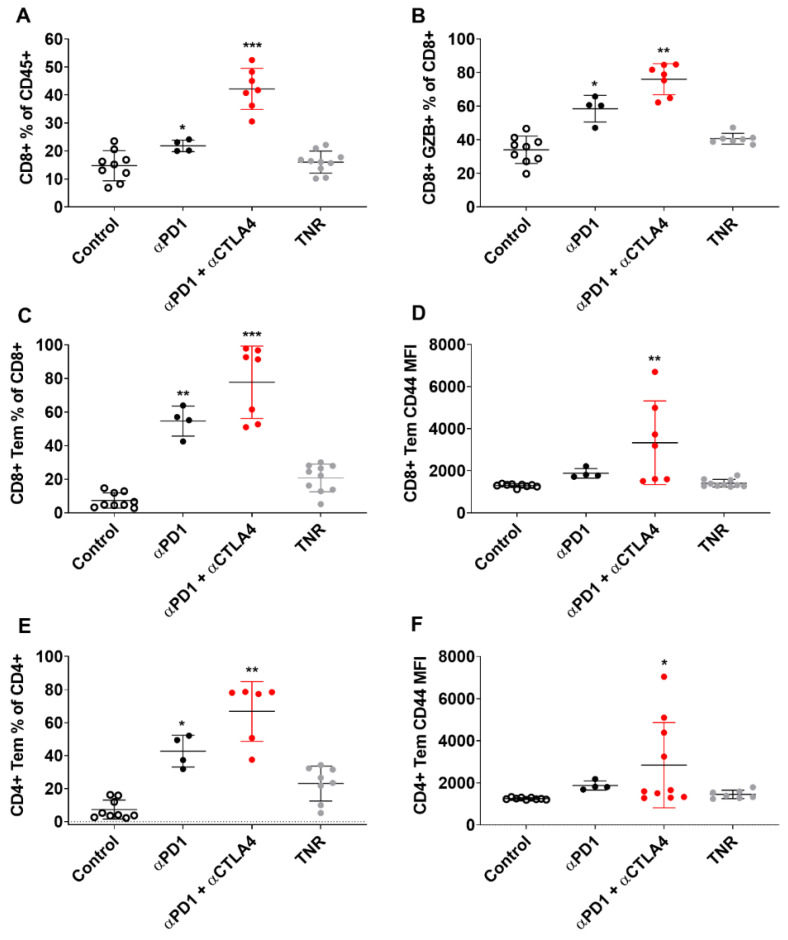
Multicolour Flow cytometry analysis of CT tumour associated immune cells after treatment. Percentages of (**A**) CD8^+^ T cells relative to CD3^+^ cells, (**B**) GZB+ CD8+ TILS relative to total CD8+ TILS, (**C**) CD8+ T_EM_ cells relative to total CD8^+^ cells (**D**) CD8+ T_EM_ cells relative to total CD8+ cells MFI (**E**) CD4+ T_EM_ cells relative to total CD4^+^ cells (**F**) CD4^+^ T_EM_ cells relative to total CD4^+^ cells MFI across all treatment arms. Data are shown as individual values with the mean ± S.D. and are representative of *n* = 5–10 mice/ group. * *p* < 0.05; ** *p* < 0.01; *** *p* < 0.001 compared to TNR.

**Figure 5:**
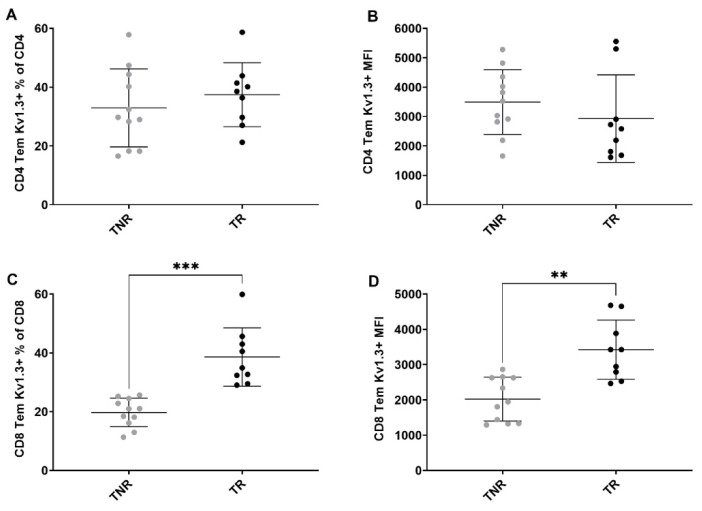
Multicolour flow cytometry analysis of tumour-associated Kv1.3-expressing T cells after treatment. Percentages of (**A**) Kv1.3 CD4^+^ T_EM_ cells relative to total CD4^+^ cells (**B**) CD4^+^ T_EM_ cells relative to total CD4^+^ cells MFI (**C**) Kv1.3 CD8^+^ T_EM_ cells relative to total CD8^+^ cells MFI (**D**) Kv1.3 CD8^+^ T_EM_ cells relative to total CD8^+^ cells MFI between TR and TNR. Data are shown as individual values with the mean ± S.D. and are representative of *n* = 8–10 mice/ group. ** *p* < 0.01; *** *p* < 0.001 TR compared to TNR.

Tumour-infiltrating immune cells were measured in each of the treatment groups and separated into TRs and TNRs (Figure 4 and Figure 5). t-SNE and Rphenoptype clustering provided a measure of the immunophenotypic changes associated with treatment response or lack of response in each treatment cohort. Rphenograph clustering identified CD8 T cells (clusters 1, 2, 11), CD4 T cells (clusters 6, 8, 17), NK cells (clusters 9, 15), CD11b myeloid cells (clusters 7, 12, 14), Ly6G-positive neutrophils (cluster 3), and SiglecF-positive eosinophils (cluster 10) (Figure S9A). The different immune cell populations associated with treatment response and treatment non-response are listed in Supplementary Table S6. The greatest differences were measured in tumour-associated CD8^+^ T cells (CD8^+^, GZB^+^ CD8^+^ and CD8^+^ T_EM_ T cells, Table 2). Separation of the t-SNE based on treatment response or lack of response in each treatment cohort revealed the strongest increase in cluster 1 in ICI-responsive tumours compared to TNRs, which corresponds to CD8^+^ T_EM_ cells (Supplementary Figure S9B). Manual gating for quantification and statistical analysis is shown in Supplementary Figure S10. CD8^+^ T_EM_ tumour infiltration as a % of CD8^+^ cells was significantly higher in αPD1 monotherapy (** *p* < 0.01) and αPD1 + CTLA4 combination therapy (** *p* < 0.01) TRs compared to TNRs.

## 4. Discussion

Immunological memory is a hallmark of adaptive immunity, antigen exposure causes naïve T cells to differentiate into effector T (T_EFF_) cells, effector memory T (T_EM_) cells, tissue-resident memory T (T_RM_) cells and central memory T (T_CM_) cells. While previous studies have shown that response to ICI therapy is positively correlated with infiltration of CD8^+^ T cells [9,13,20], the development of a robust immunological memory response is critical for durable disease control. Rapid immunological memory response is mediated by memory cells (T_EM_ and T_RM_ cells) present in tumour-draining lymph nodes, or within the tumour. Recent studies show that T_EM_ cells in particular are accurate biomarkers of response to ICI therapy [21]. Not only do responsive tumours have increased post-treatment tumour antigen-specific T_EM_ cells compared to non-responders but this specific effector memory T cell response is maintained after cessation of treatment [21]. Unlike other T memory cells, T_EM_ cells exhibit substantial upregulation of the Kv1.3 potassium channel upon activation. T_EM_ cells increase Kv1.3 expression from approximately 300 channels per cell in the resting state to 1500–2000 channels per cell in activated cells [22,23,24]. T_EM_ cells have been intensively investigated for their role in autoimmune pathology and Kv1.3 targeting agents have been developed as effective therapeutic strategies [16,22,24]. The current study has used one of these Kv1.3 targeting peptides and developed a theranostic with high affinity and specificity for Kv1.3 (Supplementary Figure S3). Our study demonstrates that radiopharmaceuticals targeting Kv1.3 can successfully stratify response to ICIs in vivo.

The CT26 syngeneic colon cancer model has been extensively characterised and response to ICIs has been shown to be mediated by infiltration of CD8^+^ T cells and a reduction in suppressive F4/80^+^ myeloid cells [9,20]. A similar profile was observed in the current study (Supplementary Table S6). Further, we assessed memory T cell populations associated with successful therapy response. No significant change in infiltrating T_CM_ or T_RM_ cells was observed; however, the ICI response was associated with significant changes in T_EM_ cell infiltration. The T_EM_ population accounts for ~7% of the TILs in control-treated tumours, ~20% in TNRs, and in responsive tumours, the % was significantly higher with 55% in αPD1 monotherapy and 78% in combined αPD1 + αCTLA4 responsive tumours. This correlated well with tumour uptake of the Kv1.3-targeting radiopharmaceutical [^18^F]AlF-NOTA-KCNA3P with significantly higher retention in tumours responsive to αPD1 or combined αPD1 + αCTLA4 therapy compared to TNRs (Table 1, Figure 3).

Care should be taken, however, with the interpretation of tracer uptake as Kv1.3 channels are also expressed in numerous cancer tissues where they are involved in cell proliferation and may be up-regulated or down-regulated depending on the cancer type and stage of the disease. Kv1.3 has been shown to be upregulated in some types of breast and colon cancer but down-regulated in others including kidney, bladder, pancreas, lung, brain, stomach and prostate cancers [25,26,27,28,29,30,31]. The Kv1.3 potassium channel is also expressed in many tissues throughout the body including B lymphocytes, macrophages, fibroblasts, brain, lung, islets, thymus, spleen, lymph nodes, and testes [32] which may contribute to the background signal.

## 5. Conclusions

Overall, the current study shows that the imaging agent [^18^F]AlF-NOTA-KCNA3P is an effective biomarker for the stratification of durable response to ICIs in a syngeneic model of colon cancer correlating well with Kv1.3 expression on tumour-infiltrating T_EM_ cells. The data presented suggest that with further development Kv1.3-targeting biomarkers may have the potential to accurately stratify durable response to immunotherapy in a clinical setting.

## Figures and Tables

**Table 1:** Table shows CT26 tumour retention of [^18^F]AlF-NOTA-KCNA3P in vivo after ICI treatment evaluated by PET/CT. Data are shown as the mean %ID/g ± S.D. of control groups, treatment responsive tumours (TR) across treatment cohorts and treatment non-responders (TNR) (*n* = 10 mice/ group; * *p* < 0.05; ** *p* < 0.01, comparing TR to TNR).

Treatment Group	[^18^F]AlF-NOTA-KCNA3P Tumour Uptake (%ID/g ± SD)
Control	0.51 ± 0.19
Treatment Responsive tumours (TR) αPD1	0.87 ± 0.15 *
αPD1 + αCTLA4	1.30 ± 0.36 **
Treatment Non-Responders (TNR)	0.53 ± 0.20

**Table 2:** Flow cytometry showing immune cell types associated with positive response to αPD1 or combined αPD1 + αCTLA4 therapy in CT26 tumour bearing mice. Percentages of T cell subpopulations across control groups, treatment responder (TR) arms, and all treatment non-responsive tumours (TNR) across all treatment arms. Data are shown as the mean % of cells ± S.D. and are representative of *n* = 5–10 mice/ group, * *p* < 0.05; ** *p* < 0.01, *** *p* < 0.001 comparing TR to TNR.

	CT26 Tumour-Infiltrating Immune Cells			
Treatment Group	CD8^+^ % of CD3^+^	GZB^+^ CD8^+^ % of CD8^+^	CD8^+^ T_EM_ % of CD8^+^	CD4^+^ T_EM_ % of CD4^+^
Control	14.74 ± 5.38	34.02 ± 8.15	7.32 ± 4.53	7.25 ± 5.75
TR αPD1	21.35 ± 2.78 *	58.46 ± 7.96 *	54.61 ± 8.96 **	42.68 ± 9.65 *
αPD1 + αCTLA4	42.13 ± 7.35 ***	76.06 ± 9.20 **	77.67 ± 21.50 ***	66.81 ± 18.09 **
TNR	16.43 ± 4.61	40.66 ± 3.20	20.69 ± 8.26	23.09 ± 10.62

## Data Availability

The data presented in this study are available on request from the corresponding author.

## Supplementary Materials

The following supporting information can be downloaded at: https://www.mdpi.com/article/10.3390/cancers14051217/s1, Table S1. Calibration data for NOTA-KCNA3P. Table S2. Mass spectrometry data of AlF-NOTA-KCNA3P. Table S3. Summary of tumour volumes in controls, ICI treatment responders (TR) and treatment non-responders (TNR). Table S4. Summary of ICI treatment responders (TR) and treatment non-responders (TNR) across all therapy arms. Table S5. Tumour growth inhibition % on day 21. Table S6. Table showing the tumour associated immune cell populations. Figure S1. Calibration curve of NOTA-KCNA3P. Figure S2. Radio- and UV chromatograms of the reformulated radiotracer, [^18^F]AlF-NOTA-KCNA3P. Figure S3. Electrophysiological studies of NOTA-KCNA3P and EgK5 on Kv1.,3, Kv1.2 and Kv1.5 channels. Figure S4. Representative time activity curves (TACs). Figure S5. Ex vivo biodistribution analysis of [^18^F]AlF-NOTA-KCNA3P. Figure S6. Western blot assessment of Kv1.3 in TRs and TNRs. Figure S7. Graph showing % intact parent [^18^F]AlF-NOTA-KCNA3P in plasma. Figure S8. Representative maximum intensity projection PET/CT images of [^18^F]AlF-NOTA-KCNA3P uptake. Figure S9. Multicolour flow cytometry analysis. Figure S10. Representative manual gating strategy for immunophenotyping of tumour samples.
