# Supplementary material for: Imaging Kv1.3 Expressing Memory T Cells as a Marker of Immunotherapy Response
Source: Cancers (Basel). 2022 Feb 26;14(5):1217. doi: 10.3390/cancers14051217 (PMC8909107; doi:10.3390/cancers14051217)
Supplement: Supplementary file 1 [file cancers-14-01217-s001.zip › cancers-1583145-supplementary.pdf]

cancers

MDPI

Supplementary

Imaging Kv1.3 Expressing Memory T Cells as a Marker of Immunotherapy Response

Julian L Goggi[1,2*]; Shivashankar Khanapur[1]; Boominathan Ramasamy[1]; Siddesh V Hartimath[1]; Jun Rong Tang[1]; Peter Cheng[1]; Yun Xuan Tan[1]; Xin Yi Yeo[3]; Jung Sangyong[2,3]; Stephanie Shee Min Goay[4]; Seow Theng Ong[4]; Youyi Hwang[5]; K George Chandy[4] and Edward G Robins[1,6]

1 Institute of Bioengineering and Bioimaging (IBB), Agency for Science, Technology and Research (A*STAR), 11 Biopolis Way, #01-02 Helios, Singapore 138667.
2 Department of Physiology, Yong Loo Lin School of Medicine, National University of Singapore, Singapore 119077
3 Institute of Molecular and Cell Biology (IMCB), A*STAR, 11 Biopolis Way, #01-02 Helios, Singapore 138667.
4 LKCMedicine-ICESing Ion Channel Platform, Lee Kong Chian School of Medicine, Nanyang Technological University, 59 Nanyang Drive, Singapore 636921.
5 Singapore Immunology Network (SIgN), A*STAR, 8A Biomedical Grove, Immunos, Singapore, 138648.
6 Clinical Imaging Research Centre (CIRC), 14 Medical Drive, #B1-01, Yong Loo Lin School of Medicine, National University of Singapore, Singapore 117599.
* Correspondence: julian_goggi@ibb.a-star.edu.sg; Tel.: (+65 6824 7093)

1.1 General information

Aluminium chloride (≥ 99.999%) and sodium fluoride were procured from Sigma-Aldrich Pte Ltd, Singapore. Glacial acetic acid and Saline solution (0.9% w/v) were purchased from JT Baker and Braun Medical Industries, respectively. All other reagents were procured from Merck, VWR chemicals and Fisher Scientific. All commercially obtained reactants and reagents were used as such without any further purification. [^{18}F]AlF-NOTA-KCNA3P synthesis was carried out in a closed Thermo Scientific™ conical reactivial™ (1 ml). Sep-Pak® light (46 mg) accell™ plus QMA carbonate (Part No.: 186004540), Oasis HLB plus light cartridge (Part No.: WT186005125) and Sep-Pak C18 plus short cartridges (Part No.: WAT020515) were purchased from Waters Pacific Pte Ltd, Singapore.

No-carrier-added (nca) aqueous [^{18}F]fluoride ion was produced by the irradiation of ^{18}O-enriched water via the [^{18}O(p,n)^{18}F] nuclear reaction using a GE PETtrace 860 cyclotron. Radiochemical purification was performed on a Knaur semi-preparative radio-HPLC system comprising of two Knaur Smartline 1050 pumps, Manual injection valve (6-port/3-channel), SmartMix 100 solvent mixer, Smartline UV-Detector 2520 and Flow-Count radio-HPLC NaI detection system. Quality control analytical radio-HPLC was performed on an UFLC Shimadzu radio-HPLC system equipped with dual wavelength UV detector and a NaI/PMT-radiodetector (Flow-Ram, LabLogic). Radioactivity measurements were made with a CRC-55tPET dose calibrator (Capintec, USA).

LC-MS 2020 (Shimadzu Asia Pacific Pte Ltd) was used for identification of the labelled peptide. The mass spectrometer was operated in electrospray positive ionization mode. The mass spectrometer settings were optimized as follows: interface voltage, 4.5 kV; nebulizer gas flow, 1.5 L / min; drying gas flow, 15 L / min; desolvation line (DL) temperature, 250° C; heat block temperature, 250° C. Other mass spectrometer parameters were tuned automatically. Mass spectral data analysis was done manually for identification of labelled peptide.

1.2 Peptide Synthesis

The NOTA-KCNA3P precursor peptide, NOTA-RTCESASH-KFEGPCLRDSNCANVCKTEGFH-GGKCKGLRRRCFCTKHC (Cys1 & Cys8, Cys2 &

Cys5, Cys3 & Cys6, Cys4 & Cys7 bridges) was custom synthesized by Chinese Peptide Company (CPC) with >95% purity.

1.3 Synthesis of [^{19}F]AlF-NOTA-KCNA3P

Aluminium chloride (2 mM) and sodium fluoride (2 mM) stock solutions (10 ml each) were prepared in 0.1M sodium acetate buffer adjusted to pH 4 with glacial acetic acid. Aliquots of the $AlCl_3$ (160 µl) and NaF (320 µl) stock solutions were added to a 1.5 ml GC vial containing NOTA-KCNA3P (0.25 mg), followed by an equal volume of ethanol (480 µl). The reaction vial was sealed and heated at 100° C for 15 min without stirring. After cooling to room temperature, the crude reaction mixture was diluted with water (10 ml) and the product trapped on an Oasis HLB plus light cartridge (30 mg). The cartridge was then washed with 5 ml of water. [^{19}F]AlF-NOTA-KCNA3P was eluted with 70% ethanol in saline (0.5 ml), and diluted with 0.9% w/v saline to a final concentration of 10% ethanol in saline.

Serial dilutions of NOTA-KCNA3P (31.25-500 µg/ml) were assessed by analytical radio-HPLC (Aeris™ 5µm PEPTIDE XB-C18, 100 Å, 250 x 4.6 mm; 1 ml / min, λ = 220 nm, Supplementary Figure S1 and Supplementary Table S1). Gradient elution was carried out using a mixture of 0.1 % aqueous trifluoroacetic acid (solvent A) and 0.1 % trifluoroacetic acid in acetonitrile (solvent B). The following gradient elution profile was used: 0.01 - 0.20 min 10 % B, 0.20 – 10.00 min 70 % B, 10.50 - 16.00 min 10 % B. The retention time of NOTA-KCNA3P was 7.5-7.6 min (Supplementary Figure S2).

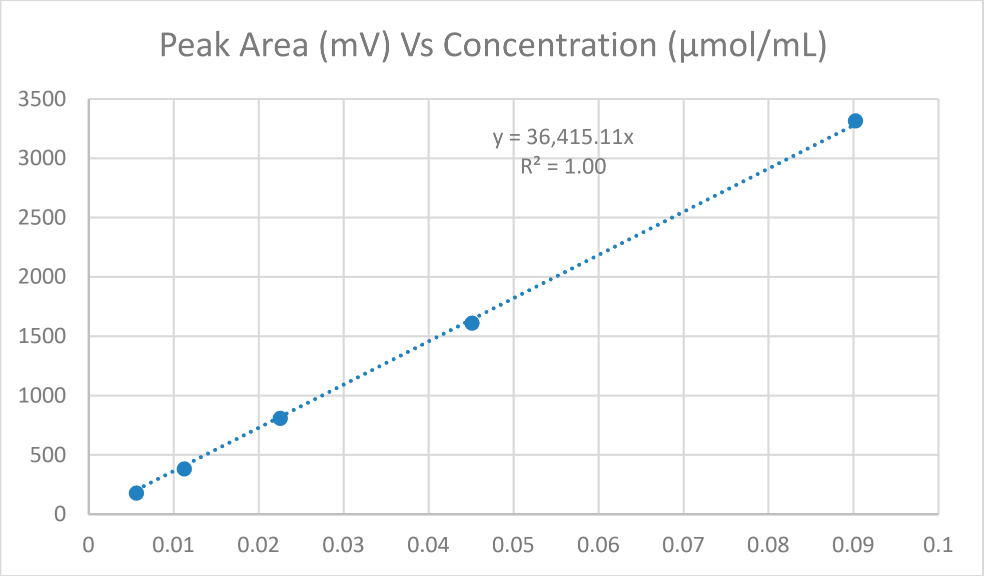

Supplementary Figure S1. Calibration curve of NOTA-KCNA3P.

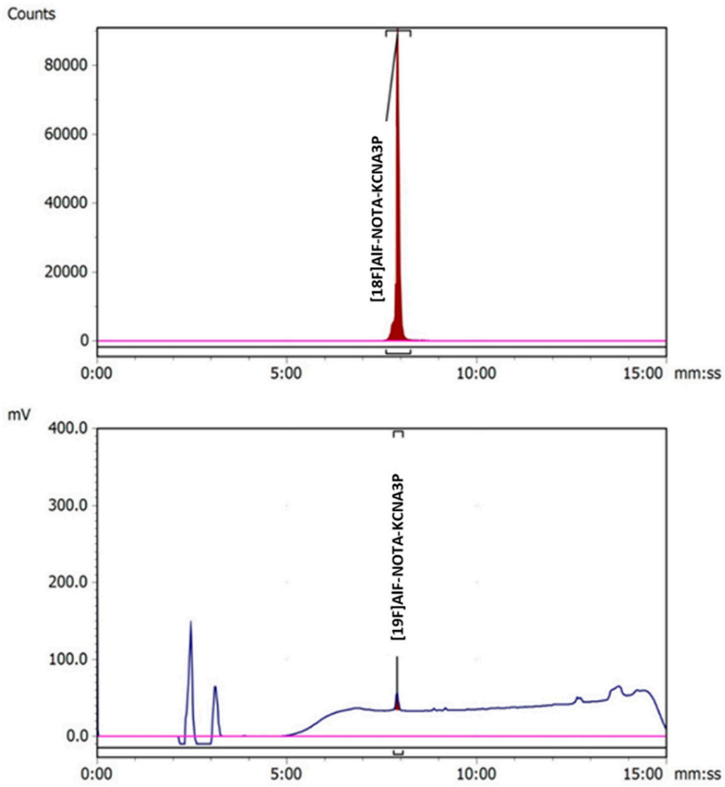

Supplementary Figure S2. Radio- and UV chromatograms of the reformulated radiotracer, [^{18}F]AlF-NOTA-KCNA3P.

1.4 Mass spectrometry characterisation of [^{19}F]AlF-NOTA-KCNA3P

After synthesis and isolation, the sample was subjected to LC-MS analysis using an Agilent Poroshell 120 EC-C18 2.7µm 4.6x50 mm; 0.4 ml / min, λ = 220 nm. Gradient elution was carried out using a mixture of 0.1 % aqueous formic acid (solvent A) and 0.1% formic acid in acetonitrile (solvent B). The following gradient elution profile was used: 0.00 – 0.01 min 10 % B, 0.01 – 8.00 min 95 % B, 8.00 – 12.00 min 95 % B, 12.00 – 12.30 min 10 % B. Liquid chromatography–mass spectrometry (LC-MS) was performed to confirm molecular mass of the [^{19}F]labelled peptide product. It was found that the mass of 5584.2 Da for [^{19}F]labelled peptide (Supplementary Table S2, Theoretical expected mass for [^{19}F]labelled peptide is 5584.4 Da).

1.5 Radiosynthesis of [^{18}F]AlF-NOTA-KCNA3P

Aqueous nca [^{18}F]fluoride (typically 10 GBq in 2.4 ml) was trapped on a Sep-Pak® light (46 mg) Accell™ plus QMA carbonate cartridge (pre-conditioned with 10 ml deionised water) and washed with a further 5 ml of water. The trapped [^{18}F]fluoride anion was then eluted with 0.9% w/v saline (0.2 ml) into a 1.5 ml Eppendorf tube. To the [^{18}F]fluoride solution was added a 24 µl aliquot of a 2 mM $AlCl_3$ stock solution prepared in 0.1 M sodium acetate buffer and pH adjusted to pH 4 using glacial acetic acid. This combined reaction mixture was transferred into a 1 ml reactor vial containing NOTA-KCNA3P (0.2-0.25 mg) followed by an equal volume of ethanol (0.2 ml). The reaction vial was then sealed and heated at 100° C for 15 min without stirring. After cooling to room temperature, the crude reaction mixture was diluted with water (3 ml) and subjected to purification by semi-preparative radio-HPLC (Aeris™ 5µm PEPTIDE XB-C18, 100 Å, 250 x 21.2 mm; 10 ml / min, λ = 254 nm). Gradient elution was carried out using a mixture of 0.1 % aqueous trifluoroacetic acid (solvent A) and 0.1 % trifluoroacetic acid in acetonitrile (solvent B). The following gradient elution profile was used: 0.01 - 0.20 min 10 % B, 0.20 -

8.00 min 70 % B, 8.00 - 10.00 min 70 % B, 11.00 - 15.00 min 10 % B. The retention time of [^{18}F]AlF-NOTA-KCNA3P was between 8.2 - 8.3 min. The collected HPLC pure fraction was then trapped on a pre-conditioned Sep-Pak Plus C18 cartridge (Preconditioning was done using 5 ml ethanol followed by 10 ml deionised water). The cartridge was then washed with 10 ml of water. [^{18}F]AlF-NOTA-KCNA3P was eluted with 70% ethanol in saline (0.5 ml), and diluted with 0.9% w/v saline to a final concentration of 10% ethanol in saline. The radiochemical purity of [^{18}F]AlF-NOTA-KCNA3P was assessed by analytical radio-HPLC (Aeris™ 5μm PEPTIDE XB-C18, 100 Å, 250 x 4.6 mm; 1 ml / min, λ = 220 nm). Gradient elution was carried out using a mixture of 0.1 % aqueous trifluoroacetic acid (solvent A) and 0.1 % trifluoroacetic acid in acetonitrile (solvent B). The following gradient elution profile was used: 0.01 - 0.20 min 10 % B, 0.20 – 10.00 min 70 % B, 10.50 - 16.00 min 10 % B. The retention time of [^{18}F]AlF-NOTA-KCNA3P was 7.5-7.6 min (Supplementary Figure S1 and S2). [^{18}F]AlF-NOTA-KCNA3P (Figure 1) was isolated with a non-decay corrected radiochemical yield of 11.9 ± 6.2 % within 45-50 min (n = 8) from aqueous [^{18}F]fluoride. The radiochemical purity was greater than 99% and molar activity was 75 ± 45 GBq / μmol at the end of the synthesis (n = 8).

1.6 Electrophysiology studies

NOTA-KCNA3P and EgK5 were prepared as 1-10 mM stock solutions in P6N buffer (10 mM sodium phosphate, 0.8% w/v, NaCl, and 0.05% v/v Tween 20, pH 6), diluted with 0.1% (w/v) BSA in external buffer. The effects of peptides on Kv1.2, Kv1.3 and Kv1.5[1] channels were evaluated by patch-clamp using a QPatch HTX automated electrophysiology platform (Sophion, Denmark) (Supplementary Figure S3). The giga-seal and whole-cell requirements for the automated electrophysiology were the following: minimum seal resistance of 0.1 GΩ, holding potential -90 mV, holding pressure -20 mbar, positioning pressure -70 mbar. Currents were elicited by 200 ms depolarizing pulses to 40 mV from holding potential -80 mV. The inter-pulse interval were 30, 45 and 10 s for Kv1.2, Kv1.3 and Kv1.5 respectively. External solution comprised in mM: 4.5 KCl, 160 NaCl, 1 $MgCl_2$, 2 $CaCl_2$, 10 HEPES; pH 7.4. Internal buffer comprised in mM: 160 KF, 2 $MgCl_2$, 10 EGTA, 10 HEPES; pH 7.2.

Prolonged exposure experiments were carried out as previously described[2]. Briefly, B82 cells expressing Kv1.2, L929 cells expressing Kv1.3 and MEL cells expressing Kv1.5 were seeded into T25 flasks for 72 h, then washed and replenished with freshly prepared media before overnight treatment with 0.1, 1 or 10 μM of NOTA-KCNA3P, or 10 μM EgK5, diluted in P6N buffer. Control cells were treated with media containing P6N buffer at the same final concentration. NOTA-KCNA3P, EgK5 and P6N were directly added into the flask with cell densities around 70% confluency. Patch clamp recordings of both control and treated cells were carried out at the end of each experiment. The average amplitudes of peak current at pulses 18-20 were measured and normalized with cell capacitance using Sophion QPatch software 5.6 and exported to Microsoft Excel and GraphPad Prism 7 for analysis. Cells with membrane capacitances of 10-30 pF (B82-Kv1.2 and L929-Kv1.3) or 3-12 pF (MEL-Kv1.5) were used for data analysis.

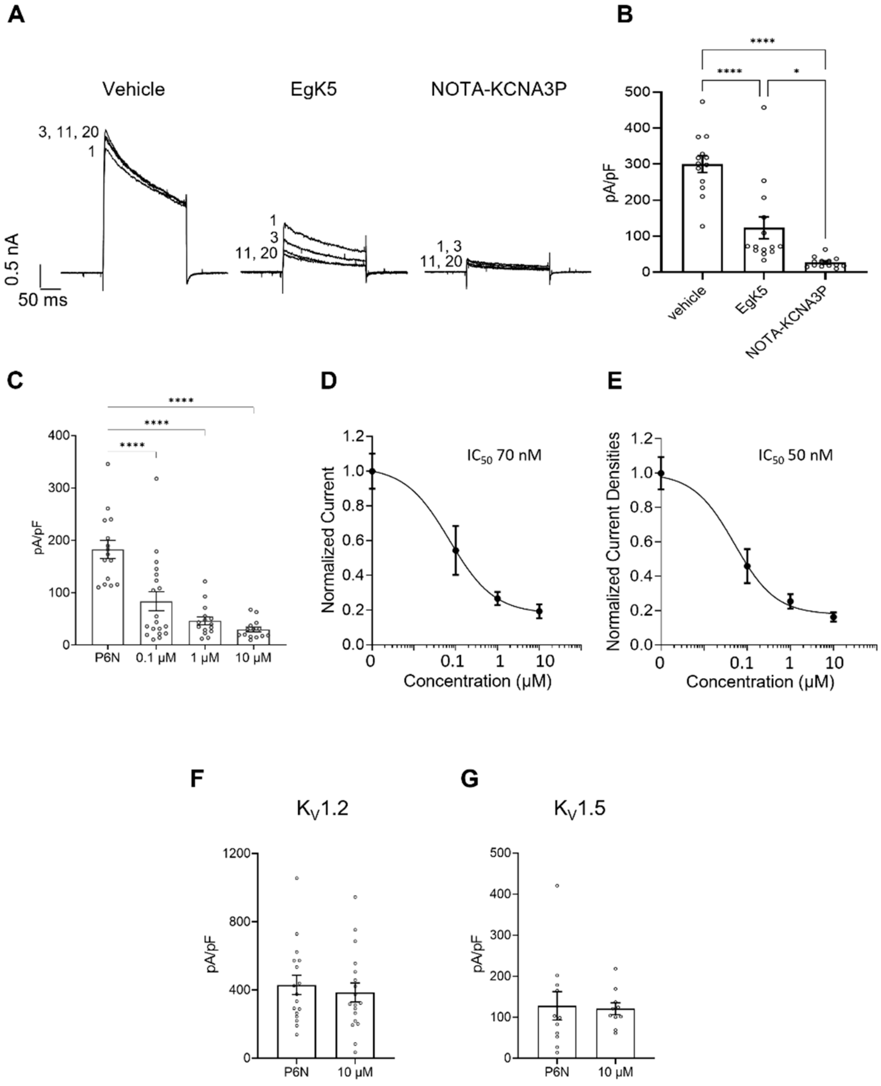

Supplementary Figure S3. A. Kv1.3 currents in cells pre-treated overnight with vehicle (control), EgK5 (10 μM) or NOTA-KCNA3P (10 μM). Current amplitude at pulses 1, 3, 11 and 20 are shown. B. Average current amplitude at pulses 18–20 normalized for membrane capacitance (pA/pF). C. NOTA-KCNA3P reduced Kv1.3 current density in a concentration-dependent manner. D-E. Kv1.3 average current amplitudes measured at pulses 18-20 and current densities (pA/pF) for each concentration were normalized to the average values of control cells and fitted into non-linear regression curve to determine IC_{50} values (50% suppression concentrations) (n = 13). F-G. Pre-treatment with NOTA-KCNA3P (10 μM) did not affect Kv1.2 and Kv1.5 currents. All bar graphs depict mean ± SEM. Statistical analysis for B and D: 1-way ANOVA, * $p< 0.05$, **** $p < 0.0001$.

1.7 In vivo biodistribution of [^{18}F]AlF-NOTA-KCNA3P

The biodistribution of [^{18}F]AlF-NOTA-KCNA3P was assessed in BalbC mice bearing CT26 tumours treated with combined PD-1 and CTLA4 therapy. The animals were imaged dynamically for 120 minutes and the distribution and excretion characteristics assessed. Overall the excretion profiles for the radiopharmaceutical shows excretion via the kidneys and hepatobiliary routes (Supplementary Figure S4). Blood clearance is rapid; muscle uptake is relatively low.

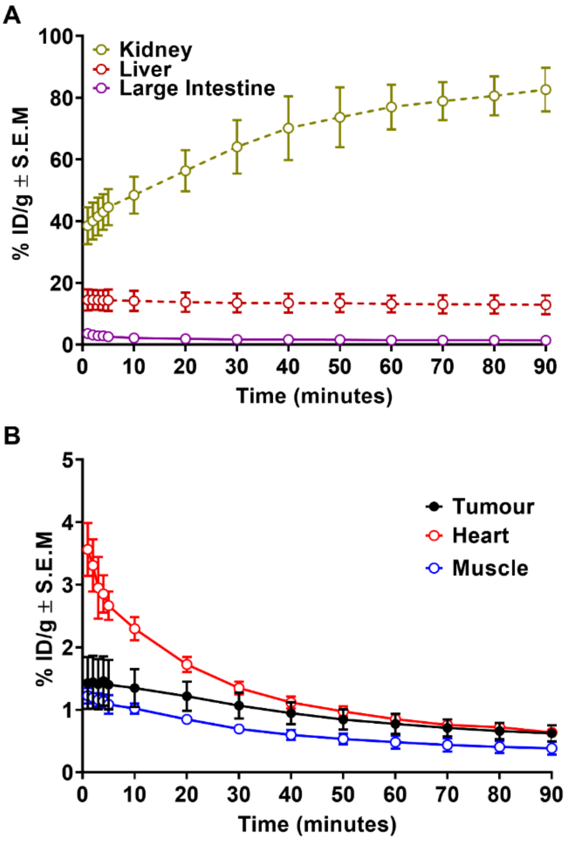

Supplementary Figure S4. Representative time activity curves (TACs) showing the biodistribution of [^{18}F]AlF-NOTA-KCNA3P in naïve CT26 tumour bearing animals (n=4). **A.** Shows TACs highlighting the excretion of the radiolabeled peptides. **B.** Shows TACs highlighting control tumour, heart and muscle uptake.

1.8 Ex vivo biodistribution

The biodistribution of [^{18}F]AlF-NOTA-KCNA3P was also assessed in BalbC mice bearing CT26 tumours treated with combined PD-1 and CTLA4 therapy by standard dissection procedures at 80 minutes post injection, when tracer kinetics were judged to be at equilibrium. The animals were injected with ~1MBq [^{18}F]AlF-NOTA-KCNA3P via the lateral tail vein and sacrificed at 80min post-injection, the tissues excised, weighed and radioactivity quantified using a Wallac gamma counter (Supplementary Figure S5).

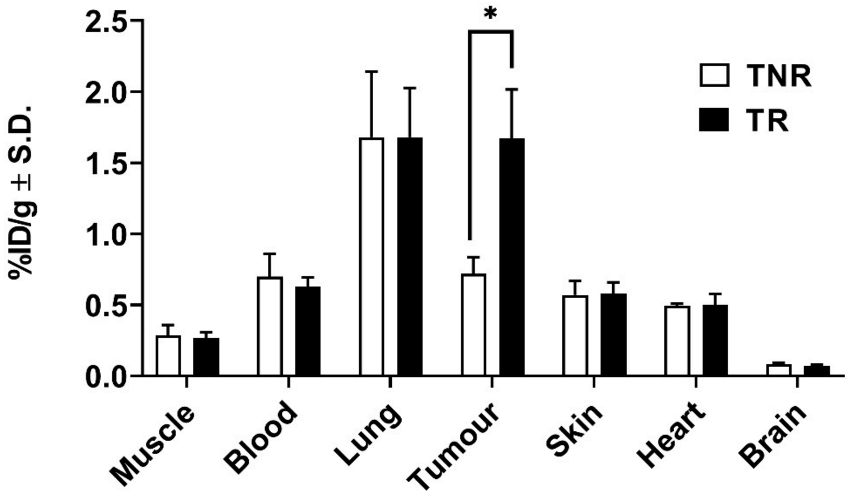

Supplementary Figure S5. Ex vivo biodistribution analysis of [18F]AlF-NOTA-KCNA3P retention in selected organs. ICI treated responder (TR, black) and treated non responder (TNR, white) animals were sacrificed 80min post-injection, tissues excised, weighed and radioactivity quantified using a Wallac gamma counter. Bars represent the mean of 5 animals ± SEM, * *P*<0.05.

1.9 Western blot analysis of Kv1.3

Tumour Kv1.3 expression on infiltrating lymphocytes (TILs) were quantified using standard western blotting procedures as previously described[3]. The following antibodies were used: anti-Kv1.3 KCNA3 (APC-101, Alomone) and anti-GAPDH (SC-47724, Santa Cruz). Kv1.3 was significantly higher on TRs compared to TNRs (Supplementary Figure S6A and B).

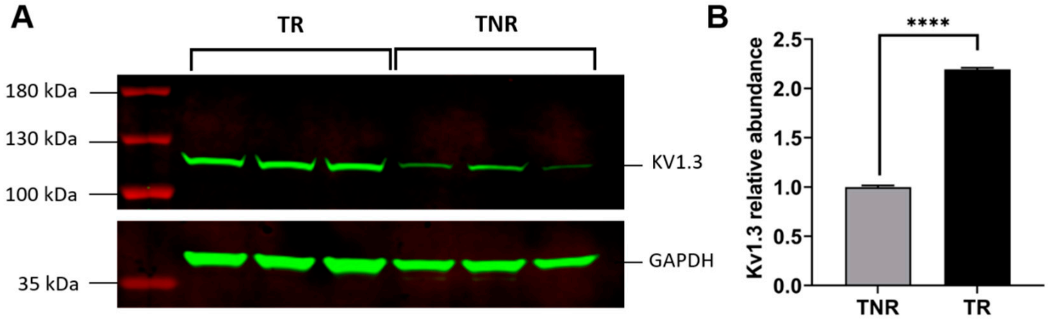

Supplementary Figure S6. (**A**.) Western blot assessment of Kv1.3 and (**B**.) Relative abundance of Kv1.3 in ICI treated responders TR (black) and TNR (grey), data are shown as mean ± S.D. and are representative of n=3 mice/ group, **** *P*<0.0001, comparing TR to TNR.

1.10 Ex vivo metabolite analysis of [18F]AlF-NOTA-KCNA3P

After intravenous injection of ~20MBq [18F]AlF-NOTA-KCNA3P, blood samples (~300 µL) were collected via the orbital plexus into heparinized polypropylene centrifuge tubes, at 0, 30, 60 and 120 min p.i. Blood samples were centrifuged at 2,500 x g for 10 min. An equal amount of 10% sulfosalicylic acid was added to the supernatant plasma samples and vortexed to precipitate out remaining proteins. The samples were centrifuged at 2,500 x g for 5 min at 4 °C, and the supernatant analyzed via radioHPLC. [18F]AlF-NOTA-

KCNA3P was shown to breakdown slowly over time with less than 30% metabolism over 120 minutes as shown in Supplementary Figure S7.

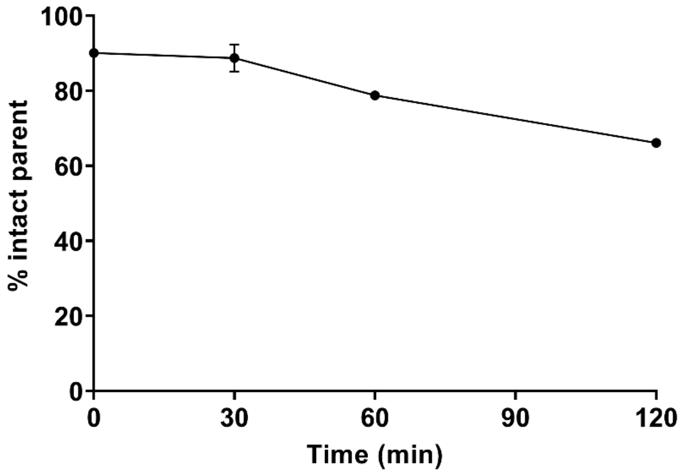

Supplementary Figure S7. Graph showing % intact parent [^{18}F]AlF-NOTA-KCNA3P in plasma (data shown as % intact parent ± SD).

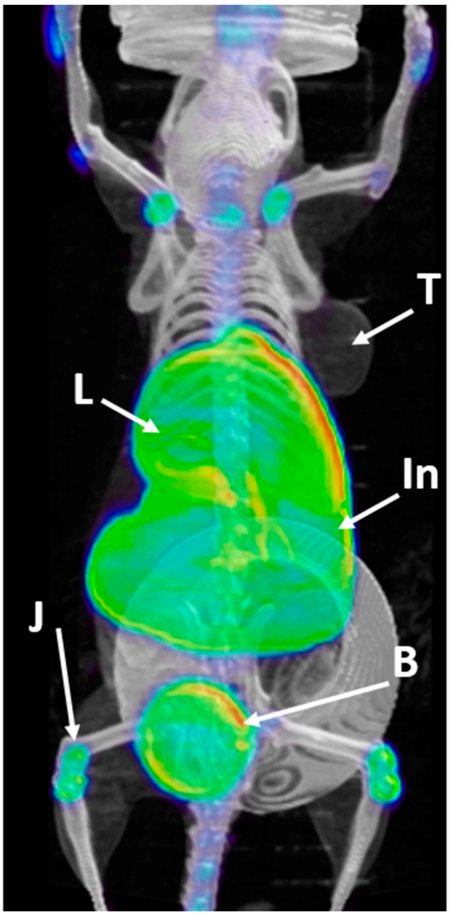

Supplementary Figure S8. Representative maximum intensity projection PET/CT images of [^{18}F]AlF-NOTA-KCNA3P tumour uptake in a CT26 tumour bearing Control treated animal showing whole body distribution for reference (white arrows show tumour (T), liver (L), intestines (In), bladder (B) and joint (J) uptake). Mice administered ~10 MBq [^{18}F]AlF-NOTA-KCNA3P, and images acquired from 60-80 mins post tracer injection.

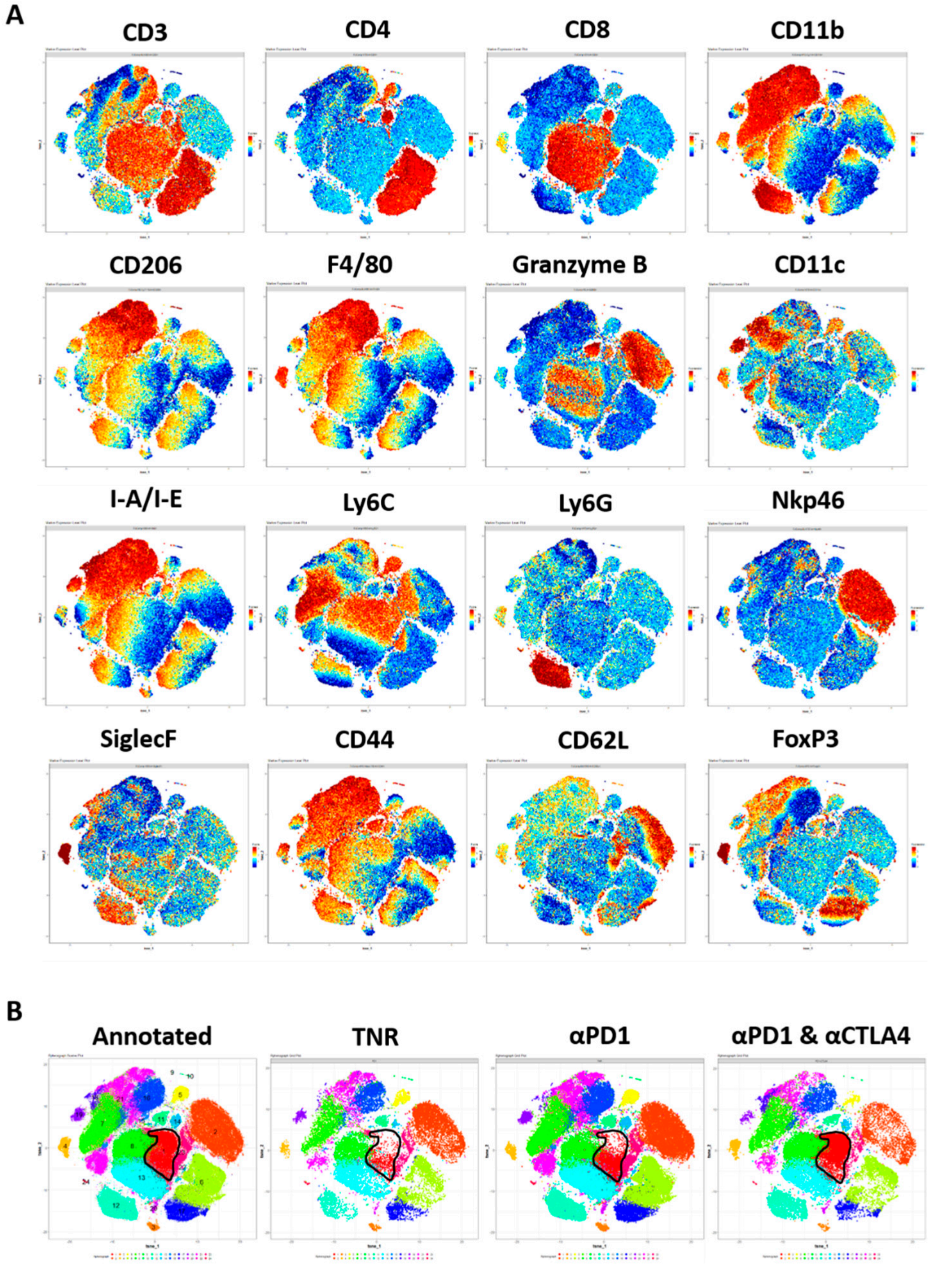

Supplementary Figure S9. Multicolour Flow cytometry analysis of immune cell profile of the tumour from CT26 tumour-bearing mice at day 14 post-induction of ICI monotherapy or combination therapies. **A.** Marker expression level plots of the markers used for Rphenograph clustering on all tumours used for the t-SNE. t-SNE plot showing unbiased Rphenograph clustering of cell populations based on the expression of CD3, CD4, CD8, CD11b, CD11c, CD206, F4/80, Granzyme B, I-A/I-E, Ly6C, Ly6G, Nkp46 and Siglec-F. **B.** tSNE plots showing the distribution of cells in the Rphenograph clusters in each treatment arm. TNR n=10, αPD1 n=5, αPD1+αCTLA4 n=7 (cluster showing Tem cells ringed in black).

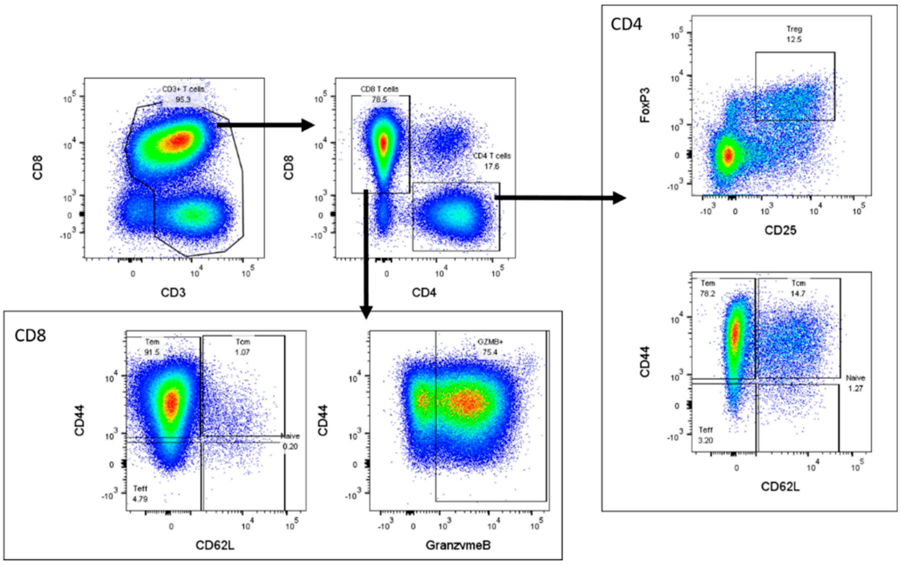

Supplementary Figure S10. Representative manual gating strategy for immunophenotyping of tumour samples. Viable hematopoietic cells were identified by size, viability stain-negative, singlet gating and CD45-positive expression as previously described [4]. Gating for $CD3^+CD4^+$ and $CD3^+CD8^+$ T cells were identified and investigated for effector subtypes.

Tables

Conc (ug/mL)	Conc (µmol/mL)	Peak area (mV)
500	0.090246192	3313.4
250	0.045123096	1608.2
125	0.022561548	805.5
62.5	0.011280774	380.5
31.25	0.005640387	176.8

Supplementary Table S1. Calibration data for NOTA-KCNA3P

Peptide	Sequence	m/z (Calculated)	m/z (observed)
**AlF-NOTA-KCNA3P**	AlF-NOTA-RTCESASHK-FEGPCLRDSNCANVCKTEGFHG GKCKGLRRRCFCTKHC (Cys1 & Cys8, Cys2 & Cys5, Cys3 & Cys6, Cys4 & Cys7 bridges)	5584.4	5584.4 [M+H], 792.4 [×7-7H = MW]

Supplementary Table S2. Mass spectrometry data of AlF-NOTA-KCNA3P

Treatment arm	Days post inoculation	CT26 tumour volume ($mm^3 \pm SD$)
**Control**	6	142.08 ± 27.05

	9	301.53 ± 61.68
	12	530.35 ± 105.13
	15	734.81 ± 206.34
	21	1252.26 ± 235
**Treatment Responders (TR)**	6	106.77 ± 21.85
**αPD1**	9	150.28 ± 58.04
	12	198.66 ± 79.97
	15	196.96 ± 70.45
	21	325.99 ± 141.27
**αPD1 + αCTLA4**	6	138.70 ± 20.55
	9	197.87 ± 47.75
	12	225.01 ± 69.21
	15	117.30 ± 53.20
	21	169.99 ± 77.22
**Treatment Non Responders**	6	143.80 ± 20.02
**(TNR)**	9	268.40 ± 41.70
	12	474.52 ± 120.36
	15	724.55 ± 346.55
	21	1660.42 ± 352.12

Supplementary Table S3. Summary of tumour volumes in controls, ICI treatment responders (TR) and treatment non-responders (TNR).

	**Treatment Responders (TR)/ Treatment Non-Responders (TNR) (No. mice)**
**ICI Treatment**	**CT26**
Control	0/8
αPD1	5/12
αPD1 + αCTLA4	10/12
% Overall Therapy Response	62.5

Supplementary Table S4. Summary of ICI treatment responders (TR) and treatment non-responders (TNR) across all therapy arms in syngeneic CT26 and MC38 colon cancer models

**Treatment arm**	**%TGI (mean ± SD)**
αPD1	80.9 ± 13.2

αPD1 + αCTLA4	97.3 ± 7.9
TNR	-36.6 ± 31.6

Supplementary Table S5. Tumour growth inhibition % on day 21 for each treatment arm compared to control.

A

	CD3⁺ % of CD45⁺	CD4⁺ % of CD3⁺	CD8⁺ % of CD3⁺	GZB⁺ % of CD45⁺	F4/80⁺ % of CD45⁺
**Control**	35.6 ± 9.0	17.2 ± 8.0	14.7 ± 5.4	24.4 ± 8.3	13.1 ± 3.9
**TR**					
**αPD1**	36.8 ± 5.7	12.2 ± 2.9	21.3 ± 2.8*	36.2 ± 3.1*	14.5 ± 2.8
**αPD1 + αCTLA4**	59.3 ± 8.8*	11.0 ± 2.4	42.1 ± 7.4**	44.6 ± 8.6**	5.1 ± 2.6*
**TNR**	36.7 ± 7.0	15.4 ± 6.2	16.4 ± 4.6	25.6 ± 6.7	14.2 ± 3.7

B

	CD4⁺ naïve % of CD4⁺	CD4 Teff % of CD4⁺	CD4 Tcm % of CD4⁺	CD4 Tem % of CD4⁺	CD4⁺ Treg % of CD4⁺
**Control**	13.8 ± 2.2	72.5 ± 10.3	2.0 ± 1.8	7.2 ± 5.7	7.2 ± 2.5
**TR**					
**αPD1**	7.3 ± 2.5	26.0 ± 9.2	16.1 ± 4.2*	42.7 ± 9.7*	14.4 ± 3.9
**αPD1 + αCTLA4**	6.0 ± 4.0	29.2 ± 11.7	15.8 ± 3.6*	66.8 ± 18.1**	13.5 ± 4.8
**TNR**	9.6 ± 3.2	47.9 ± 18.1	6.5 ± 3.3	23.1 ± 10.6	10.8 ± 2.6

C

	CD8⁺ naïve % of CD8⁺	CD8 Teff % of CD8⁺	CD8 Tcm % of CD8⁺	CD8 Tem % of CD8⁺	CD8⁺ GZB⁺ % of CD8⁺
**Control**	11.9 ± 6.6	76.3 ± 8.3	0.6 ± 0.5	7.3 ± 4.5	34.0 ± 8.2
**TR**					
**αPD1**	3.8 ± 0.9	32.6 ± 18.1	0.8 ± 0.5	54.6 ± 9.0**	58.5 ± 8.0*
**αPD1 + αCTLA4**	0.5 ± 0.4**	29.4 ± 15.7	0.7 ± 0.4	77.7 ± 21.5***	76.1 ± 9.2**
**TNR**	7.6 ± 5.6	55.1 ± 15.5	0.7 ± 0.4	20.7 ± 8.3	40.7 ± 3.2

Supplementary Table S6. Table showing the tumour associated immune cell populations from CT26 tumour-bearing mice at day 12 post-induction of αPD1 monotherapy or combination therapies. **A**. Percentages of $CD3^+$, $CD4^+$, $CD8^+$, GZB^+ and F4/80⁺ immune cell subpopulations are shown across control groups, treatment responders (TR) and treatment non-responders (TNR) across all treatment arms. **B**. Percentages of $CD4^+$ naïve, $CD4^+$ T effector, $CD4^+$ T central memory, $CD4^+$ T effector memory and $CD4^+$ T regulatory immune cell subpopulations are shown across control groups, treatment responders (TR) and treatment non-responders (TNR) across all treatment arms. **C**. Percentages of $CD8^+$ naïve, $CD8^+$ T effector, $CD8^+$ T central memory, $CD8^+$ T effector memory and $CD8^+$ GZB^+ immune cell subpopulations are shown across control groups, treatment responders (TR) and treatment non-responders (TNR) across all treatment arms. Data are shown as mean % of cells ± S.D. and are representative of n=5-10 mice/ group, * P<0.05; ** P<0.01, *** P<0.001, comparing TR to TNR.

References

1. Grissmer, S.; Nguyen, A.N.; Aiyar, J.; Hanson, D.C.; Mather, R.J.; Gutman, G.A.; Karmilowicz, M.J.; Auperin, D.D.; Chandy, K.G. Pharmacological characterization of five cloned voltage-gated K+ channels, types Kv1.1, 1.2, 1.3, 1.5, and 3.1, stably expressed in mammalian cell lines. *Mol Pharmacol* **1994**, *45*, 1227-1234.
2. Ong, S.T.; Bajaj, S.; Tanner, M.R.; Chang, S.C.; Krishnarjuna, B.; Ng, X.R.; Morales, R.A.V.; Chen, M.W.; Luo, D.; Patel, D.; et al. Modulation of Lymphocyte Potassium Channel KV1.3 by Membrane-Penetrating, Joint-Targeting Immunomodulatory Plant Defensin. *ACS Pharmacol Transl Sci* **2020**, *3*, 720-736, doi:10.1021/acsptsci.0c00035.
3. Bielanska, J.; Hernandez-Losa, J.; Perez-Verdaguer, M.; Moline, T.; Somoza, R.; Ramon, Y.C.S.; Condom, E.; Ferreres, J.C.; Felipe, A. Voltage-dependent potassium channels Kv1.3 and Kv1.5 in human cancer. *Curr Cancer Drug Targets* **2009**, *9*, 904-914, doi:10.2174/156800909790192400.
4. Goggi, J.L.; Hartimath, S.V.; Xuan, T.Y.; Khanapur, S.; Jieu, B.; Chin, H.X.; Ramasamy, B.; Cheng, P.; Rong, T.J.; Fong, Y.F.; et al. Granzyme B PET Imaging of Combined Chemotherapy and Immune Checkpoint Inhibitor Therapy in Colon Cancer. *Molecular imaging and biology : MIB : the official publication of the Academy of Molecular Imaging* **2021**, doi:10.1007/s11307-021-01596-y.
